# Physical Exercise as a Multimodal Tool for COVID-19: Could It Be Used as a Preventive Strategy?

**DOI:** 10.3390/ijerph17228496

**Published:** 2020-11-17

**Authors:** Diego Fernández-Lázaro, Jerónimo J. González-Bernal, Nerea Sánchez-Serrano, Lourdes Jiménez Navascués, Ana Ascaso-del-Río, Juan Mielgo-Ayuso

**Affiliations:** 1 Department of Cellular Biology, Histology and Pharmacology, Faculty of Health Sciences, Campus of Soria, University of Valladolid, 42003 Soria, Spain; 2 Neurobiology Research Group, Faculty of Medicine, University of Valladolid, 47005 Valladolid, Spain; 3 Cavidito Research Group, Department of Health Sciences, University of Burgos, 09001 Burgos, Spain; jejavier@ubu.es; 4 Microbiology Unit of the Santa Bárbara Hospital, Castilla-Léon Health (SACyL), 42003 Soria, Spain; nsanchezser@saludcastillayleon.es; 5 Department of Nursing, Faculty of Health Sciences, Campus of Soria, University of Valladolid, 42003 Soria, Spain; lourdes.jimenez@uva.es; 6 Clinical Pharmacology Service, IdISSC, San Carlos Clinical Hospital, 28040 Madrid, Spain; ana.ascaso@salud.madrid.org; 7 Department of Biochemistry, Molecular Biology and Physiology, Faculty of Health Sciences, Campus of Soria, University of Valladolid, 42003 Soria, Spain; juanfrancisco.mielgo@uva.es

**Keywords:** COVID-19, physical exercise, health care, immune system, inflammation, oxidative stress, nitric oxide

## Abstract

The severe acute respiratory syndrome coronavirus 2 (SARS-CoV-2) or coronavirus disease 2019 (COVID-19) is a novel coronavirus not previously recognized in humans until late 2019. On 31 December 2019, a cluster of cases of pneumonia of unspecified etiology was reported to the World Health Organization in China. The availability of adequate SARS-CoV-2 drugs is also limited, and the efficacy and safety of these drugs for COVID-2019 pneumonia patients need to be assessed by further clinical trials. For these reasons, there is a need for other strategies against COVID-19 that are capable of prevention and treatment. Physical exercise has proven to be an effective therapy for most chronic diseases and microbial infections with preventive/therapeutic benefits, considering that exercise involves primary immunological mediators and/or anti-inflammatory properties. This review aimed to provide an insight into how the implementation of a physical exercise program against COVID-19 may be a useful complementary tool for prevention, which can also enhance recovery, improve quality of life, and provide immune protection against SARS-CoV-2 virus infection in the long term. In summary, physical exercise training exerts immunomodulatory effects, controls the viral gateway, modulates inflammation, stimulates nitric oxide synthesis pathways, and establishes control over oxidative stress.

## 1. Introduction

### 1.1. Origin of COVID-19

Acute viral respiratory infections are essential public health trouble, with high morbidity and mortality in the world. Coronavirus (CoV) viral pathogens are a considerable family of viruses that cause illnesses ranging from the common cold to more severe diseases, such as Middle East Respiratory Syndrome (MERS-CoV) and Severe Acute Respiratory Syndrome (SARS) [1]. The severe acute respiratory syndrome coronavirus 2 (SARS-CoV-2) or coronavirus disease 2019 (COVID-19) is a novel coronavirus not previously recognized in humans until late 2019. On 31 December 2019, a cluster of bouts of pneumonia of unspecified etiology came to the attention of the World Health Organization (WHO) in Wuhan, China. SARS-CoV-2 was identified by the Coronavirus Study Group of the International Committee on Virus Taxonomy in affected patients [2].

### 1.2. Respiratory Clinical Symptoms

A large number of patients show flu-like symptoms and recover at home [3]. However, the most significant concern is for patients who develop a severe condition associated with respiratory difficulties and pneumonia. Pneumonia represents approximately 20% of patients infected by this new coronavirus, and 5% of patients require critical care, characterized by the presence of respiratory failure, severe acute respiratory syndrome, renal failure, septic shock, and multi-organ failure. These patients must be admitted to the hospital or even to intensive care units to increase their chances of survival [4].

### 1.3. Strategies to Fight COVID-19

The availability of safe and effective drugs to treat the infection COVID-19 causes remains limited and needs to be assessed by extra clinical trials [5]. In this line, drugs regularly use clinical assistance, such as neuraminidase inhibitors (oseltamivir, paramivir, and zanamivir), and antiviral agents (ganciclovir, acyclovir, and ribavirin) are not practical for SARS-CoV-2. Drugs possibly useful for 2019-nCoV include: remdesivir, lopinavir/ritonavir, lopinavir/ritonavir combined with interferon-β, convalescent plasma, and monoclonal antibodies [6]. Chloroquine phosphate, an anti-malarial drug, its efficacy, and safety against COVID-19 respiratory disease also appear to be satisfactory in recent clinical trials in China in treating patients infected by SARS-CoV-2 [7].

However, there is a need for other strategies against COVID-19 that are competent in prevention and treatment. Physical exercise (Ex) (planned structured and repetitive activity performed with purpose) has proven to be an effective therapy for most chronic diseases, with preventive/therapeutic benefits and considering the primary immunological mediators involved [8]. Even an Ex-induced shift in immune response may be dependable for improved survival after respiratory virus infection [9]. Exercise’s immune response effects accumulate over time and form the immunological adaptations in both systems (innate and adaptive), and these often work in conjunction with the overall immune response [10]. During incubation and non-severe stages, the adaptive immune response must remove the SARS-CoV-2 and avoid disease progression to severe stages. Innate immune cells also need to recognize the invasion of the virus, often by pathogen-associated molecular patterns [11]. Other potential effects of Ex have been described that could help control COVID-19, such as attenuation of the inflammatory response [12], modulation of oxidative stress [13], and increase in nitric oxide (NO) synthesis [14]. Therefore, Ex may confer protection against COVID-19 by enhancing the functioning of some physiological systems. This insight may help to design the adequate physical exercise multimodal tool that is preventive and/or therapeutic against COVID-19.

## 2. Intervention through Physical Exercise on the Immune Function in COVID-19

### 2.1. Interferon Modulation by Physical Exercise

COVID-19 virus is inhaled and binds to non-specific receptors on the respiratory epithelium, such as intercellular adhesion molecule 1 (ICAM-1), that permit the cell to become infected. The pathogen-associated molecular patterns (PAMPs) on SARS-CoV-2 are likely to be recognized by Toll-like receptors (TLR) 2, 3, and 4, initiating a rapid innate immune response against viral invasion [15,16]. PAMPs-TLRs interaction stimulates production of the interferon regulatory factor (IRF), which in turn produces interferon (IFN) type I (IFN-α and IFN-β), and subsequent binding to the IFN receptor gives rise to the expression of a diversity of interferon-stimulated genes (ISGs), with outcome antiviral and immunomodulatory effects to control and destroy the virus disease [17,18]. However, the viral proteins involved in the modulation of this hosting type I IFN response are E, M, N (structural proteins), and ORF (non-structural), which inhibit IFN signaling by downregulating janus kinase/signal transducers and the activators of transcription (JAK/STAT) pathway, concretely decreasing STAT1 phosphorylation [19].

The resistance Ex training elicits similar mitogen-activated protein kinase (MAPK) activation in resistance-trained men with significant elevations in STAT1 phosphorylation [20,21,22]. The activation MAPK could partly restore the mechanism of inhibition established by the viral infection of COVID-19, and probably continue a downstream cascade that culminates in control of SARS-CoV-2. IFN I also activates p38 MAPK to induce gene transcription for the antiviral response [23]. Ex stimulates p38 [24], which, in addition to the effects on the muscle, could promote antiviral and anti-proliferative effects specific to IFN type I resulting from p38 signaling.

Regular Ex modulates another novel INF pathway, with a probable COVID-19 prevention activity. Ex is beneficial for maintaining the improvement of the immune status, associated with a rise in IFN-2 (γ) in the plasma [25]. Thus, regular moderate Ex seems to modulate its release and increase its levels to those necessary for the human body to maintain good health [26]. Recently, Kang et al. [27] have described the antiviral properties of IFN-γ with its ability to block the virus’s entry into the extracellular and intracellular phases of replication. The pathways of action of IFN-γ are diverse: alteration of the niche of replication, stopping of the process of gene expression, unstructuring the virus by breaking the assembly of the nucleocapsid, and prevention of viral reactivation by inhibiting the transcription of a master regulator of the virus. This mechanism is to accentuate the direct antiviral pathways of IFN-γ that may be stimulated by Ex practice [26]. Effective innate immune response is associated with IFN-α, IFN-β, and IFN-γ, and may play a role in a protective or destructive response against COVID-19 [28]. Therefore, physical exercise could be a tool, through the immune system, that modulates the reactions of the INF pathway that could control viral replication and induce a more adequate immune response.

### 2.2. Modulation of the Response to Viral Infection by Innate Immune Cells through Physical Exercise

The expression of PAMPs by immune and tissue cells provides the host with the ability to detect and respond to infection by viruses by resistance to viral replication in all cells, induction of apoptotic cell death in infected cells, increased major histocompatibility complex (MHC) class I expression to enhance antigen presentation, activation of dendritic cells (DCs) and macrophages, and stimulation of natural killer (NK) cells to improve their activity [29]. SARS-CoV-2 has mechanisms to evade PAMPs-mediated responses or to subvert these pathways actively (which should be investigated), which could reduce the innate antiviral immune response that triggers severe states in patients [29].

The neutrophils, innate immune cells, respond to exercise-derived stimuli. The chemotaxis and phagocytosis increase after moderate activity Ex (50% maximal oxygen uptake (VO_2_max)) but not in strenuous Ex (80% VO_2_max), where neutrophils oxidative activity is attenuated [30]. Monocyte cells are mobilized with moderate duration (<60 min) and intensity (<60% VO_2_max) Ex [10]. Both non-lymphocyte cells (monocytes and phagocytes) present phenotypes related to: (i) effector or cytotoxic functions and differentiated or mature (CD16^+^ monocytes and CD16^−^ neutrophils); and (ii) integrin and intracellular adhesion molecules, and a range of chemokine receptors, such as CCR5, CCR6, CXCR1, CXCR2, CXCR3, and CXCR4, that have ligands for activated endothelium and permit tissue migration [31]. Lower et al. [9] exercised mice using a motorized treadmill at a speed of 8–12 min at a 1 and 5% grade for 30 min. The treadmill speed was approximately 55–65% of VO_2_max [9]. In this study [9], this increased the expression of IL-4 and the eotaxins. The eotaxins act as a chemoattractant for eosinophils with granules [32]. The granules contain abundant ribonucleases that degrade single-stranded RNA viruses [32], such as SARS-CoV-2. Moreover, Qin et al. [33] have demonstrated a pronounced lymphopenia (decrease CD3^+^) in COVID-19. However, Ex stimulates natural killer T (NKT) cells, which have a regulatory effect depending on IL-4 and considerably increase two-fold by Ex [9]. NKT cells are a broad group of CD3^+^ T cells co-expressing the T cell antigen receptor (TCR) and NK cell markers [34]. The cell trafficking in response to Ex could be a strategy of boosting the innate immune response [26,30] required to eliminate COVID-19 and to prevent the advance of the virus.

NK cells have various mechanisms to kill virus-infected cells, such as increased expression of extracellular death receptors and the release of granules with cytolytic actions by exocytosis [35]. Still, respiratory viruses (influenza virus and respiratory syncytial virus) have evolved a mechanism to evade NK cell response [36]. Ex plays a role in increasing the NK cell number and subset distribution and functional capability of NK cells at the individual cell level [30]. Forty-five minutes of high (80% VO_2_max) and moderate (50% VO_2_max) intensity treadmill Ex was associated with significant shifts in circulating proportions of NK cells during and 2 h after Ex [37]. A study on sixteen healthy cyclists also concluded that Ex (3 × 30 min cycling at −5%, +5%, and +15% of lactate threshold) evokes a preferential redeployment of NK cell subsets with a high differentiation phenotype [38]. This phenotype leads to increased surface expression of inhibitory killer immunoglobulin-like receptor (KIR) molecules as CD158b and augments NK cell cytotoxic activity (NKCA) [38,39], which could improve the degree of immunity to SARS-CoV-2. Another mechanism capable of inducing virus death by apoptosis of NK cells and CD8^+^ effector T cells is through direct interaction between the surface Fas receptor and the FasL ligand [40]. FasL was stimulated in day three post-infection of the influenza virus in mice that practiced moderate Ex (55–65% VO_2_max) [9]. An Ex immune program may positively modulate cytotoxic NK cell and CD8^+^ activity in blood [30,38,39], and may be a therapeutic preventive intervention to help combat SARS-CoV-2.

Furthermore, in post-exercise or recovery periods, some leukocytes (mainly monocytes and lymphocytes) mobilize between tissues and blood. Monocytes and lymphocyte leucocytes are overexpressing adreno-receptors (β2-ARs) and glucocorticoid receptors, which improves their sensitivity to catecholamines and/or cortisol [41]. This indicates that a mobilization/response is mediated by activation of the sympathetic nervous system and the hypothalamus pituitary adrenal (HPA) axis, which are strongly activated by Ex [42]. This redistribution of the leukocytes may control the SARS-CoV-2 infection in early periods of the disease.

### 2.3. Modulation of the Adaptive Immune Response to Viral Infection through Physical Exercise

Helper T cells orchestrate the overall adaptive immune response, but initially, PAMP signaling activates the dendritic cells. Dendritic cells’ cytokines (CCL3, CXCL9, and CXCL10), generated by antigen presenting cells in the respiratory tract, dictate the direction of T cell responses to the viral infection [43]. T cells response, CD4^+^, and CD8^+^ can clear the virus and protect the host from lethal diseases or respiratory illnesses, such as influenza A and para-influenza virus [44]. However, when comparing healthy individuals to critically ill patients in the acute phase of SARS, 80% presented lymphopenia with a significantly alarming decrease between 80–100% of CD4^+^ and CD8^+^ [45]. The dysregulation of the adaptive immune response in the severe patient group with COVID-19 in Wuhan (China) was probably a pronounced decrease of CD4^+^, CD8^+^, and regulatory T cells [33], which hastens the exceptional production of proinflammatory cytokines [1]. The senescence in T cells (inverted CD4^+^/CD8^+^ T cell ratio; increased frequency and proportion of senescent T cells) increases infection susceptibility to novel pathogens [10], such as COVID-19. Regular Ex may facilitate the selective apoptosis of these senescent T cells and stimulate T cells’ replacement [26,30].

The new T cells are capable of responding to novel antigens because they expand the naıve T cell repertoire, alleviate symptoms, and produce biomarkers associated with immunosenescence and the immune risk profile (IRP) [46]. In 102 healthy non-smoker males with 43.0 ± 0.6 VO_2_max average, this was associated with a lower proportion of senescence and a higher proportion of naïve cells in CD8^+^ T cells [47]. These may signify that acceptable levels of aerobic status could alone utilize preserving effects on the aging immune system. A healthy lifestyle with practiced regular physical exercise may decrease the risk of host infection. Therefore, Ex could represent the safest and least expensive immunotherapy treatment [48].

Moreover, IL-7 therapy could enhance immune responses in patients with limited naïve T cell numbers, as in aged patients or after disease-induced or iatrogenic T cell depletion [49]. SARS-CoV-2 induces dysregulation and a lack of T cells [11]. In this sense, by immunoblotting and real-time reverse transcription polymerase chain reaction (RT-PCR), the IL-7 expression was confirmed with both protein and mRNA increased levels by regular moderate Ex [50], which could stimulate thymus function when IL-7 increases in plasma. Ex could improve the immune response of T cells, through IL-7, in patients with deficiencies in their defenses, who are more susceptible to severe stages of COVID-19.

The activation and proliferation of CD8^+^ T cells against SARS-CoV were of higher frequency and intensity than CD4^+^ T cells. Still, both T cell responses were essential to control viral infection [19]. Conversely, a decreasing trend in T cell proliferation during and after Ex has been reported, suggesting a compromise of immune function. Migration of CD4^+^ and CD8^+^ T cells (ex vivo) reduced to supernatants of human rhinovirus (HRV)-infected bronchial epithelial cell line 1 h after completing a 2 h of running with an intensity of 60% VO_2_max [51]. However, some T cells (CD4^+^, CD8^+^, and γδ T cells) with a higher migration capacity and more efficient power appear to fall during Ex recovery and may have gone into the peripheral blood earlier [25]. Concretely, Simpson et al. [52] have observed T cells’ activation and proliferation after exercise. T cells increased significantly after 30 min in a moderate state, following their viral peptides’ stimulation against common viral antigens, such as cytomegalovirus (CMV) and Epstein–Barr virus (EBV). Many of the expanded T cell clones are also specific for the CMV and EBV antigen [42]. In this way, Ex may be an immune enhancer able to recognize the antigen that caused the initial response and destroy any infected cell.

### 2.4. Impact of Physical Exercise on the Humoral Immune Response

B cells develop an essential immune response activity to fight respiratory viral infections such as SARS-CoV-2 [53]. Contact between CD4+ T cells and naive B cells in secondary lymphoid tissues results in their proliferation and antibody class-switching, with neutralizing virus-specific antibodies crucial for optimal viral clearance [53]. B cell subsets with phenotypes characteristic of naive, non-isotype switched memory cells and antibody-secreting cells accumulate in CoVs. Thus, humoral immunity is essential to control the continuous phase of CoV infection [53].

The information on seroconversion for SARS-CoV has shown peak specific IgM at day nine after disease onset and the switching to IgG by week two [54]. In patients infected with COVID-19, antibody responses to SARS-CoV-2 in 285 patients with COVID-19 were within 19 days after symptom onset, with 100% of patients testing positive for antiviral IgG. Seroconversion for IgG and IgM occurred simultaneously or sequentially. Both IgG and IgM titers plateaued within six days after seroconversion [55]. Therefore, the immune system generates its defense strategy against SARS-CoV-2 by secreting antibodies by antigenic activation. These antibodies neutralize the ability of the virus to infect and identify them to facilitate their elimination [54,55].

In past epidemics, two strategies of immunization have been employed. The antigen stimulation of MERS-CoV infection by using the specific 9-mer epitope within the S1 protein (CYSSLILDY) generated the highest B cell antigenicity plot, and can form the most considerable number of interactions of MHCI alleles [56]. Subsets of antibodies, called neutralizing antibodies (NAbs), reduce the viral load by binding to the epitopes of the viral particles’ external surface, thus blocking entry of the virus into the cells and viral replication. These have proven useful in several viral infections, such as MERS-CoV, SARS, Chikungunya, Ebola, and Zika virus infections [57].

In this context, Ex might have a function, as immuno-enhancing exploited this natural Ex stress-response to augment immunization efficacy. Ex builds a local inflammatory response that gives rise to natural stress that boost immunization efficacy. Ex increases the production and presence of antibodies against the vaccine strain in the serum and the memory T cell response to antigen stimulation [10,30].

Ex practice has modulated positive plasticity of the immune system [26,30]. A study [58] with 10-month aerobic exercise training showed antibody titers to the H1N1 and H3N2 strains of influenza A virus enhanced in older adults immunized with a trivalent influenza vaccine. Similarly, in different population groups, moderate-intensity Ex improved the response against vaccine strains (influenza, tetanus toxoid, diphtheria, pneumococcal, and meningococcal). However, it caused a significantly more inadequate immune response in the non-exercising group [59]. Finally, in a randomized trial in risk groups susceptible to respiratory virus infections, a 24-week program of moderate cardio-vascular aerobic training was found to significantly increase the seroprotection of subjects from those who only stretched their muscles after receiving the influenza vaccine [60].

In the future, when the COIVID-19 vaccine will be developed, individuals who exercise continuously and regularly may develop higher antibody titers to the SARS-CoV-2 strain contained in the vaccine compared to individuals who do not exercise.

## 3. Angiotensin 2 Converting Enzyme (ACE2) in COVID-19: What Role Could Physical Exercise Play?

The renin-angiotensin system (RAS) plays an essential role in maintaining homeostasis blood pressure. The angiotensin-converting enzyme (ACE) is involved in producing angiotensin II (Ang II), so the activity of ACE triggers vasoconstriction [61]. The ACE-Ang II-AT1 receptor (R) pathway represents the RAS axis and the ACE2-Ang 1-7-Mas receptor (R)-based pathway represents the counter-regulatory RAS axis. The RAS axis, whose effects increase sympathetic nervous system tension, causes vasoconstriction, increases blood pressure, and promotes inflammation, fibrosis, and myocardial hypertrophy [62]. However, ACE2 generates Ang 1-9 from Ang I’s hydrolysis, which is then split by ACE, resulting in Ang 1-7. Ang 1-7 binds to a specific receptor, MasR, which is also a G-protein-coupled receptor (counter-regulatory RAS axis), and triggers anti-inflammatory, anti-fibrotic, and anti-proliferative actions [63]. Thus, in the RAS system, the same component can produce opposite physiological effects through different pathways [62].

Acute respiratory distress syndrome (ARDS) is the most severe form of acute lung injury. Interestingly, SARS-CoV uses ACE2 as an essential receptor for cell fusion and infections in vivo. Therefore, the downregulation of ACE2 expression in SARS-CoV infection may play a causal role in SARS’ pathogenesis, which provides a reasonable explanation for the progression of SARS patients into ARDS [64]. However, a recent study has shown that ACE2 protects the murine lungs from acute lung injury as well as SARS-spike protein-mediated lung injury [65]. These results suggest a dual role for ACE2 in SARS infections and protection against ARDS.

When the protective immune response is impaired or inadequate, the virus will increase and destroy the affected cells, especially in tissues/organs that have high expression of ACE2 (lung, heart, kidney, brain, and intestinal epithelial cells), which serves as the main entry point into cells for SARS-CoV-2 [66]. SARS-CoV-2 infection dysregulates the natural balance between the ACE2-Ang 1-7-Mas receptor axis and the ACE-Ang II-AT1 receptor pathway, with a subsequent high risk of severe consequences after exposure [67]. In this way, it allows development of severe lung damage, respiratory complications, and reduced survival rates. Additionally, COVID-19 can develop gastrointestinal disorders by permeabilizing the intestine wall, which favors the development of endotoxemia [62].

Nunes-Silva et al. have showed that physical Ex could stimulate the ACE2-Ang 1-7-MasR axis in parallel with the inhibition of the ACE-Ang II-AT1 receptor pathway [61] by activation of mechanism expression of microRNA. Thus, the activation of the ACE2-Ang 1-7-Mas receptor axis may have a role as a possible preventive mechanism to COVID-19 infection [67].

Besides, ACE2-Ang 1-7-Mas receptor axis stimulation could likely reduce post-COVID-19 cardiopulmonary sequelae, because physical exercise has been reported to reduce pulmonary fibrosis by inhibiting the TGF-ß1 signaling pathway [67]. This could be a recovery tool for patients who were infected with SARS-CoV-2. On balance, Ex may partially counteract the detrimental effect of SARS-CoV-2 binding to the ECA2 receptor by reducing the inflammatory response, creating a anti-fibrosis effect and better skeletal muscle response, promoting renal and cardiovascular protection, and improving central nervous system reflexes [61].

## 4. Effects of Physical Exercise on the Inflammatory Profile in COVID-19 Patients: Is It Useful as a Modulator of Cytokine Release Syndrome in COVID-19?

Three key factors contribute to the pathogenesis of COVID-19: excessive inflammation, immune system depression/inhibition, and a set of proinflammatory cytokines [68]. In the early stages of coronavirus invasion, some cytokines that are proinflammatory are expressed as IL-1β, IL-2, IL-6, IL-8, both IFN-α/β, tumor necrosis factor (TNFα), and three on the occasion of CeC (CCL3), CCL5, CCL2, and IP-10. These inflammatory mediators control the immune system, mainly in these initial stages by dendritic cells and activated epithelial cells [43,56]. Thus, the overproduction of these cytokines and chemokines may contribute to developing diseases that cause lung damage and fatal respiratory complications [69].

In this way, evidence suggests that cytokine release syndrome (CRS) produces an uncontrolled and overwhelming release of proinflammatory mediators, including IL-6, IL-1β, IL-10, and TNF-α, induced protein 10 (IP10), and monocyte chemoattractant protein 1 (MCP-1), which were significantly elevated in patients with COVID-19. Some were seen more often in severe patients than in non-severe patients [70,71]. The immunological mechanism of CRS is due to the delayed kinetics of virus clearance. In the initial phase, SARS-CoV evades recognition receptors and antagonizes the IFN type I response in the respiratory tract, and alveolar epithelial cells produce rapid viral replication [19,23]. The cytokine storm probably down-regulates innate and adaptive immunity against SARS-CoV-2 infection [69].

Anti-inflammatory cytokines after an acute Ex episode may also contribute to the reduction of systemic inflammation caused by CRS [72]. IL-6 released from skeletal muscle during Ex (more powerful mediator of acute-phase Ex response) results in a subsequent increase in IL-10 and IL-1 receptor antagonists (IL-1ra), which are considered anti-inflammatory agents [73]. IL-6 appears to be the significant contributor to Ex’s anti-inflammatory effects. Human skeletal muscles’ contraction produces and releases substantial amounts of IL-6 into the circulation to mobilize energy substrates similar to stress hormones [74]. Hormones released during Ex have an anti-inflammatory effect because cortisol acts as an anti-inflammatory mediator, and adrenaline regulates the production of the inflammatory cytokines IL-1β and TNF-α [30]. In summary, we believe Ex training may confer attenuating the CRS because changes in these proinflammatory cytokines may be modulated by anti-inflammatory cytokines, such as IL-1ra, IL-6, and IL-10, and cytokine inhibitors, such as cortisol, prostaglandin E2, and soluble receptors against TNF and IL-2 [26,30].

Other pathways of the anti-inflammatory response are the regulation of the expression of proinflammatory TLRs after completion of concurrent exercise programs (aerobic plus strength) [75]. Specifically, they decrease the expression of TLR4 on the surface of monocytes and macrophages, allowing control of inflation states in patients with chronic diseases, such as diabetes and/or obesity [71]. Another effect that Ex has on macrophage cells is the possibility of stimulating the transformation of inflammatory macrophages (M1) to anti-inflammatory ones (M2) [76]. This isotype change makes it possible to reduce the infiltration of macrophages into the fatty tissue, which could reduce the synthesis of inflammatory cytokines [76].

Furthermore, Ex reduced the expression of TLR4 and NF-κB, suggesting an anti-inflammatory response. It probably originates from the blockage of NF-κB translocation to the cell nucleus, resulting in murine models of neuroinflammation [77]. In this regard, exercised mice (8–12 min of moderate aerobic Ex for four consecutive days) infected with influenza virus showed significantly elevated levels of soluble TNF receptors in lung cells without modifications in TNF-α, which could trigger an inflammation control response [9]. Ex may be a medicine tool to help lower the risk of a cytokine storm in COVID-19 infection and minimize the sequela during the inflammatory condition.

## 5. The Behavior of Nitric Oxide in SARS-CoV-2 during Physical Exercise

The biological stimuli of INF up-regulate the nitric oxide inducible (iNOD) up-regulation during infection in activated cells as human airway epithelial cells and alveolar macrophages [78]. Nitric oxide (NO) is an important molecule that plays a role in neurotransmission, vasodilation, and immune responses [14]. The antimicrobial activity of NO has been described for several bacteria, protozoa, and some viruses, such as CoV [79]. However, the SARS-CoV proteins involved in the modulation of this hosting type I IFN response inhibit IFN signaling [19], which would alter the expression levels of iNOD and the production of NO [78]. Physical Ex training leads to increased blood flow and stress, which contributes to the endothelial expression of nitric oxide endothelial (eNOS). This enzyme is calcium-dependent, constitutively expressed, and releases NO [14]. Thus, Ex practice could partly restore the mechanism of control of COVID-19 by NO-releasing.

The inhibitory effect of NO on SARS-CoV infection was demonstrated in Vero E6 cells. NO inhibits viral protein, replication cycle, and RNA synthesis [78]. Åkerström et al. [78] have described that NO inhibits SARS-CoV replication by two distinct mechanisms: (i) NO or its derivatives cause a reduction in nascent expressed protein (S) palmitoylation, which affects the fusion between the S protein and its cognate receptor, the angiotensin-converting enzyme 2; (ii) NO or its derivatives cause a reduction in viral RNA production in the early stages of viral replication, and this could be due to an effect on one or both of the cysteine proteases encoded in the Orf-1a of SARS-CoV.

Ex therapy to stimulate the production of NO could be used for post-infection recovery of COVID-19 by alleviating lung damage because of its potent and selective pulmonary vasodilation [80]. Nowadays, inhaled NO is applied to treat pulmonary hypertension, ARDS, and other respiratory diseases with a relatively good safety profile [81].

## 6. Oxidative Stress Modulation through Physical Exercise of Oxidative Stress in Lung Cells Caused by COVID-19

Endogenous oxidants are formed when the lung becomes infected with bacteria or viruses and when inflammation occurs in response to physiological defense mechanisms. The lungs also receive all of the cardiac debit by exposure to oxidative sources in the blood, further increasing the lung’s oxidative stress by increasing reactive oxygen species (ROS). ROS has been observed to increase significantly in circumstances such as adult respiratory distress syndrome. Finally, severe lung injury makes mechanical ventilation with high concentrations of inspired oxygen mandatory, which further contributes to tissue damage by increasing oxygen radicals’ production [82].

SARS-CoV-2 infection triggers massive production of ROS, and excessive oxidative damage is responsible for secretion of the cytokine storm, impaired immunity, and the emergence of pulmonary dysfunction in response to COVID-19 infectio [83].

Oxidative stress (OS) occurs when protective antioxidant mechanisms do not react adequately due to defects in the antioxidant enzyme systems and increased ROS. ROS maintains inflammation and a high degree of OS by stimulating the NF-κB pathways and regulating the production of inflammatory energy, and is involved in cellular damage, including lung cells [12]. Against this background, Ex may be useful because it has been described to suppress the expression of TLR4 and NF-κB [77] and stimulate the promotion of the antioxidant response by activation of Nrf2 transcription; it may neutralize these harmful effects related to ROS [84]. Optimization of redox status through Ex may reduce oxidative stress, stimulate immunity, and reduce the adverse clinical effects of COVID-19 infection in the population.

## 7. Summary

Ex training exerts immunoregulatory effects, controls the viral gateway, modulates inflammation, stimulates NO production pathways, and establishes control over OS. Adaptation to usual Ex appears to affect immune function, particularly innate and adaptive immunity, and improve humoral immunity with increased vaccination responses. Ex may at least partially counteract the detrimental effect of SARS-CoV-2 binding to the ECA2 receptor. Ex training can activate anti-inflammatory signaling pathways. In this regard, the release of anti-inflammatory cytokines from skeletal muscle contraction, cortisol elevations, prostaglandin E2, and soluble receptors against TNF and IL-2, and increased mobilization of immunoregulatory leukocyte subtypes may be relevant in attenuating the CRS in COVID-19. Ex may enhance alternative routes of NO production, stimulating eNO with antiviral effects and post-infection lung recovery of COVID-19. The control of OS and ROS production, which produce cell damage, is modulated by the practice of physical activity by two mechanisms, the inhibition of NF-κB and the stimulation of Nrf2 pathways.

## 8. Perspectives

The prospects of Ex against COVID-19 infection, outlined in this manuscript, lead us to believe that the implementation of Ex programs appropriate to individuals is a useful complementary tool for prevention, enhancing recovery, improving quality of life, and providing immune protection in the long term. The intensity should be adjusted to the patient’s current situation and previous sports history. Therefore, Ex programs should be individualized.

## Abbreviations

Coronavirus (CoV), Middle East Respiratory Syndrome (MERS-CoV), Severe Acute Respiratory Syndrome (SARS), severe acute respiratory syndrome coronavirus 2 (SARS-CoV-2), coronavirus disease 2019 (COVID-19), World Health Organization (WHO), physical exercise (Ex), nitric oxide (NO), intercellular adhesion molecule 1 (ICAM-1), pathogen-associated molecular patterns (PAMPs), Toll-like receptors (TLRs), interferon regulatory factor (IRF), interferon (IFN), interferon-stimulated genes (ISGs), Janus kinase/signal transducers and activators of transcription (JAK/STAT), mitogen-activated protein kinase (MAPK), major histocompatibility complex (MHC), dendritic cells (DCs), natural killer (NK), maximal oxygen uptake (VO_2_max), natural killer T (NKT), T cell antigen receptor (TCR), inhibitory killer immunoglobulin-like receptor (KIR), NK-cell cytotoxic activity (NKCA), adreno-receptors (ARs), hypothalamus-pituitary-adrenal (HPA), immune risk profile (IRP), real-time reverse transcription polymerase chain reaction (RT-PCR), cytomegalovirus (CMV), Epstein–Barr virus (EBV), renin-angiotensin system (RAS), angiotensin-converting enzyme (ACE), acute respiratory distress syndrome (ARDS), cytokine release syndrome (CRS), induced protein 10 (IP10), monocyte chemoattractant protein 1 (MCP-1), IL-1 receptor antagonist (IL-1ra), nitric oxide inducible (iNOD), increasing reactive oxygen species (ROS), and oxidative stress (OS).
